# During the COVID-19 pandemic 20 000 prostate cancer diagnoses were missed in England

**DOI:** 10.1111/bju.16305

**Published:** 2024-02-27

**Authors:** Agnieszka Lemanska, Colm Andrews, Louis Fisher, Seb Bacon, Amir Mehrkar, Peter Inglesby, Simon Davy, Ben Goldacre, Brian MacKenna, Membership of the OpenSAFELY Collaborative, Alex J. Walker, Brian MacKenna, Peter Inglesby, Christopher T. Rentsch, Helen J. Curtis, Caroline E. Morton, Jessica Morley, Amir Mehrkar, Seb Bacon, George Hickman, Chris Bates, Richard Croker, David Evans, Tom Ward, Jonathan Cockburn, Simon Davy, Krishnan Bhaskaran, Anna Schultze, Elizabeth J. Williamson, William J. Hulme, Helen I. McDonald, Laurie Tomlinson, Rohini Mathur, Rosalind M. Eggo, Kevin Wing, Angel Y. S. Wong, Harriet Forbes, John Tazare, John Parry, Frank Hester, Sam Harper, Ian J. Douglas, Stephen J. W. Evans, Liam Smeeth, Ben Goldacre, Alex J. Walker

**Affiliations:** 1 Faculty of Health and Medical Sciences, University of Surrey, Guildford, UK; 2 Bennett Institute for Applied Data Science, Nuffield Department of Primary Care Health Sciences, University of Oxford, Oxford, UK

**Keywords:** COVID-19, prostate cancer incidence, prostate cancer prevalence, missed diagnosis, prostate cancer cohort, prostate cancer diagnosis

## Abstract

**Objectives:**

To investigate the effect of the COVID-19 pandemic on prostate cancer incidence, prevalence, and mortality in England.

**Patients and Methods:**

With the approval of NHS England and using the OpenSAFELY-TPP dataset of 24 million patients, we undertook a cohort study of men diagnosed with prostate cancer. We visualised monthly rates in prostate cancer incidence, prevalence, and mortality per 100 000 adult men from January 2015 to July 2023. To assess the effect of the pandemic, we used generalised linear models and the pre-pandemic data to predict the expected rates from March 2020 as if the pandemic had not occurred. The 95% confidence intervals (CIs) of the predicted values were used to estimate the significance of the difference between the predicted and observed rates.

**Results:**

In 2020, there was a drop in recorded incidence by 4772 (31%) cases (15 550 vs 20 322; 95% CI 19 241–21 403). In 2021, the incidence started to recover, and the drop was 3148 cases (18%, 17 950 vs 21 098; 95% CI 19 740–22 456). By 2022, the incidence returned to the levels that would be expected. During the pandemic, the age at diagnosis shifted towards older men. In 2020, the average age was 71.6 (95% CI 71.5–71.8) years, in 2021 it was 71.8 (95% CI 71.7–72.0) years as compared to 71.3 (95% CI 71.1–71.4) years in 2019.

**Conclusions:**

Given that our dataset represents 40% of the population, we estimate that proportionally the pandemic led to 20 000 missed prostate cancer diagnoses in England alone. The increase in incidence recorded in 2023 was not enough to account for the missed cases. The prevalence of prostate cancer remained lower throughout the pandemic than expected. As the recovery efforts continue, healthcare should focus on finding the men who were affected. The research should focus on investigating the potential harms to men diagnosed at older age.

## Introduction

During the COVID-19 pandemic, the resources, and the attention in healthcare systems globally, shifted towards preventing and managing COVID-19 [1,2]. Access to the non-COVID-19-related healthcare services changed [3], waiting times increased [4], and cancer pathways including treatment standards were adapted [5–8]. In addition, patients’ healthcare-seeking behaviour changed as people adopted social distancing (limiting face-to-face contact) and shielding (safeguarding high-risk people) to protect themselves and others from an infection, and healthcare systems from the unprecedented pressures of the pandemic [9]. This resulted in a widespread negative effect of the COVID-19 pandemic on cancer care including delays and missed opportunities in diagnoses and treatments [4–8,10,11]. The extent to which this will affect health and survival of patients is not yet fully understood. However, the effect may still be relevant for many years to come.

Early cancer diagnosis is key to positive cancer-related outcomes and long-term survival [10]. Primary care, which was severely affected by the pandemic, plays an important role in the diagnostic cancer pathway. In the early stages of the pandemic, studies in the UK and globally reported a suspension of cancer screening services [12] and reductions (of up to 84%) in urgent referrals from primary care for suspected cancers [12]. This unprecedented disruption in the diagnostic pathway of asymptomatic patients (screening) and symptomatic patients (referrals) has been shown to have a direct knock-on effect on the numbers of people being diagnosed.

A regional study in South East London from January 2019 to September 2020 showed a reduction of 18% in overall cancer diagnosis, with the majority (51%) of that accounted for prostate cancers [13]. A national study in England showed a 30% reduction in prostate cancer diagnosis between March and December 2020 as compared to the same period in 2019 [14]. The same study also showed that this reduction disproportionately affected younger men in the early stages of disease, with the characteristics of population shifting towards older men with a more advanced disease. Another England-based study ending in March 2021 [15], confirmed that the reduction affected younger men more than older men. In this study, we used a nationally representative database to understand the scale of the impact on prostate cancer incidence and prevalence in England. As we enter the recovery phases of the pandemic, we used this near real-time resource to investigate the effect and the speed of the recovery up to July 2023. The objectives were as follows:

1. To access nationally representative, near real-time data on prostate cancer incidence and prevalence via the OpenSAFELY platform from January 2015 to July 2023.
2. To analyse the counts and rates in prostate cancer incidence, prevalence, and mortality over time, assess the effect of the pandemic and speed of the recovery using the significance of the difference between the observed and predicted values.
3. To assess the difference in the demographics of the cohort, including age, ethnicity, and socioeconomic status from before to after the pandemic.

## Patients and Methods

### Study Purpose

This study was undertaken to analyse the effect of the COVID-19 pandemic on prostate cancer incidence, prevalence, and mortality in England.

### Study Design

This was a cohort study of people with prostate cancer. We used the OpenSAFELY-TPP dataset that comprised 24 million electronic healthcare records (EHRs) for people registered with primary care practices managed by the software provider, and EHR vendor, TPP [16]. OpenSAFELY-TPP covers over 40% of the English population and is nationally representative.

### Data Source

All data were linked, stored, and analysed securely within the OpenSAFELY platform (opensafely.org). Data included pseudonymised data such as coded diagnoses, medications, and physiological parameters. No free text was available. OpenSAFELY is a data analytics platform created by our team on behalf of NHS England to address urgent COVID-19 research questions. It provides a secure software interface allowing the analysis of pseudonymised EHRs in near real-time within the EHR vendor’s highly secure data centre, avoiding the need for large volumes of potentially disclosive patient data to be transferred off-site. This, in addition to other technical and organisational controls, minimises the risk of re-identification. Similarly, pseudonymised datasets from other data providers are securely provided to TPP and linked to primary care data. Further details on information governance of OpenSAFELY can be found in the Information governance section of this manuscript.

### Study Population and Study Dates

The study population were adult men. We analysed information on prostate cancer incidence, prevalence, and mortality between 1 January 2015 and 31 July 2023. In the UK, the pandemic-related restrictions started in March 2020. Therefore, to analyse the effect of the COVID-19 pandemic, we modelled the period from before the pandemic (1 January 2015–29 February 2020) to predict the data during and after the pandemic (1 March 2020–31 July 2023).

### Outcome Measures

Prostate cancer incidence was defined as the first time that a clinical code representing prostate cancer diagnosis was entered in a primary care record. Prevalence was defined as any person living with prostate cancer and registered with a TPP practice. Mortality was defined as any death that could be related to prostate cancer (having a prostate cancer code on a death certificate as primary or secondary cause). We analysed trends over time in monthly rates and annual counts. Rates were per 100 000 alive and registered adult men. We analysed demographics of the cohort by age at diagnosis, ethnicity, and socioeconomic status (represented by the index of multiple deprivation [IMD]). Counts of patients and healthcare services were rounded to the nearest 5 to comply with the rules for preventing statistical disclosure. Outcome measures were extracted from primary care data using the systematised nomenclature of medicine clinical terminology (SNOMED CT) system.

### Statistical Analysis

The observed monthly rates were visualised between 1 January 2015 and 31 July 2023. Data from before the pandemic were used to predict monthly rates and yearly counts in incidence and prevalence that would be expected during and after the pandemic as if the pandemic had not occurred. Generalised linear models and interrupted time series approach was used. The mortality data were only available from February 2019 and therefore, were not modelled. To include change in healthcare services over time in the model, the time was fitted as a continuous variable. The incidence in 2018 was unusually high (this is explored in more detail in the Discussion Section) and therefore these data were not included in the prediction. This was done not to overestimate the predicted rates, and in turn limit the risk of overestimating the effect of the COVID-19 pandemic.

The differences between the observed and predicted annual counts were calculated and presented as the percentage change from the observed. The 95% CIs of the predicted values were used to estimate the significance of the difference between the predicted and observed values (to estimate the effect of the COVID-19 pandemic).

Changes in the demographic characteristics including age at diagnosis, ethnicity and IMD were assessed year on year. The differences between the average age at diagnosis was assessed with the *t*-test and the differences between age, ethnicity and IMD categories were assessed with the chi-square test.

### Software and Reproducibility

Data management was performed using Python 3.8, with analysis carried out using Python and R. Software for data extraction and analysis, and code lists used to define variables are available for review and re-use from: https://github.com/opensafely/ProstateCancerPrevalence. Guidelines for REporting of studies Conducted using Observational Routinely-collected health Data (RECORD) were followed [17].

## Results

### Study Population

There were 285 160 participants (prevalence) in the study period. In participants for whom ethnicity was recorded, 224 280 (95%) were White, 4660 (2%) were Black and 3725 (2%) were Asian. Ethnicity was not recorded for 50 245 (18%) of study participants. In all, 33 255 (12%) were from the IMD quintile 1 (most deprived) and 69 460 (25%) were from IMD quintile 5 (least deprived) with 4620 (2%) IMD data missing. The average prevalence in the study period was 169 921 (SD 20 450) and the prevalence rate per 100 000 adult men was 1754 (133).

There were 165 410 men diagnosed in the study period (incidence), their average age at diagnosis was 71.4 years (SD 9.3; 95% CI 71.3–71.4). On average, in the dataset, there were 1575 (SD 270) men recorded as diagnosed every month, and this translated into an average rate of 16 (SD 2) diagnoses per month per 100 000 adult men. There were ~10 000 000 registered adult men in the dataset at any given time.

### The Effect of COVID-19 on Monthly Rates in Incidence, Prevalence, and Mortality

**Fig. 1:**
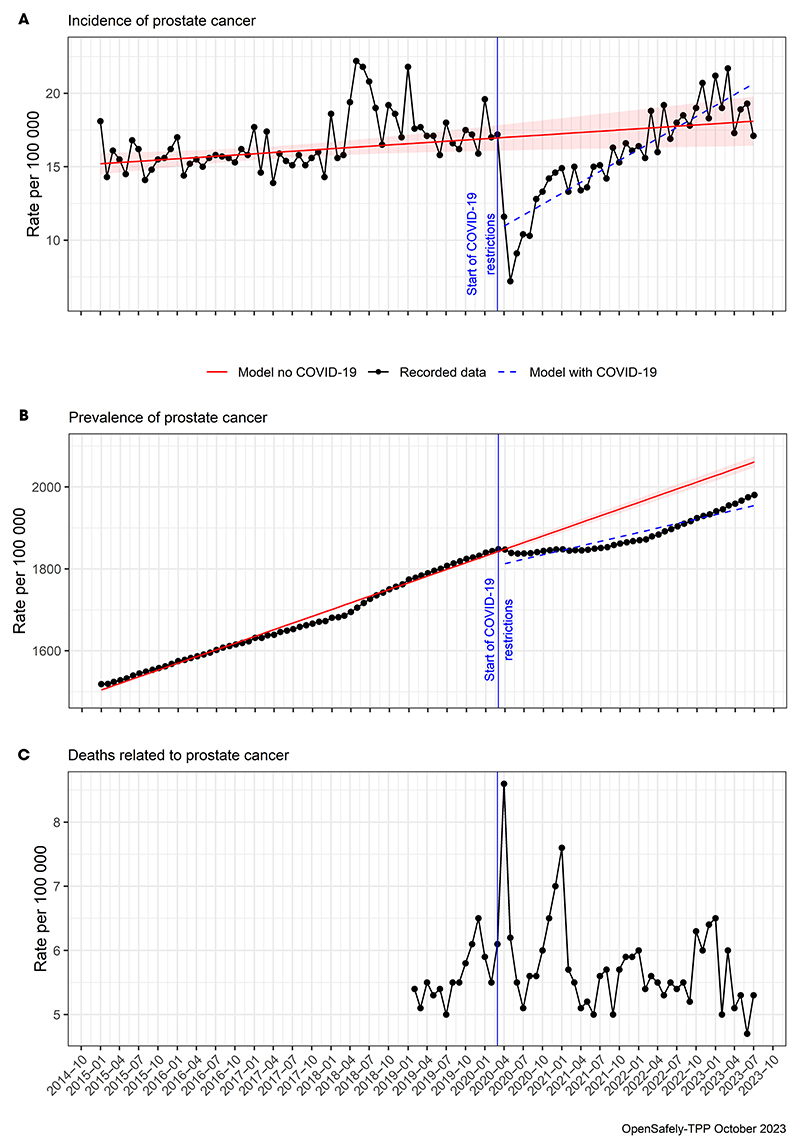
Observed and predicted trends over time from January 2015 to July 2023 showing rates per 100 000 registered adult men (**A**) prostate cancer incidence, (**B**) prevalence, and (**C**) deaths. Vertical blue line (March 2020) indicates the start of the COVID-19 restrictions. Red line represents a model (with 95% CIs) of predicted data as if the pandemic had not occurred. Mortality data were only available from February 2019, and they were not modelled.

As presented in Fig. 1A, monthly rates in incidence started to drop immediately and sharply in March 2020 from ~17 diagnoses per 100 000 to as low as seven per 100 000 in May 2020. This corresponded to a drop in monthly counts from ~1700 per month in 2019 to 700 in May 2020, with 1000 potentially missed diagnoses in May 2020 alone. From this dip in May 2020, the rates started to recover over 2020 and 2021, reaching the predicted level in 2022 and exceeding it in 2023.

The prevalence of prostate cancer was also immediately affected by the drop in incidence that started in March 2020 (Fig. 1B). Before the pandemic, there was a steady increase in prevalence rates from ~1500 per 100 000 men in 2015, through 1800 in 2019, reaching rates of ~1850 in March 2020. This upward trend stalled in March 2020, and the prevalence rates remained <1850 throughout 2020 and for most of 2021. From 2022, the prevalence rates regained its upward trajectory reaching rates of nearly 2000 at the end of the study. This was lower by ~50 men per 100 000 than the prevalence rates of >2050 predicted for the end of the study.

Figure 1C presents prostate cancer-related mortality and shows that this was generally not affected by the COVID-19 pandemic and maintained steady rates of between five and six deaths per 100 000 men over the study period. However, there were two clear peaks in mortality that could potentially be related to COVID-19, one in April 2020 and another one in January 2021, when the prostate cancer-related mortality rates increased to 8.5 and 7.5 deaths per 100 000, respectively. It is possible that these two peaks coincided with the two national lockdowns in the UK, the first one which was from March 2020 to June 2020, and the second one which was in November 2020.

### Incidence and Demographic Characteristics by Study Year

The information on incidence by study year (observed rates, observed, and predicted counts and percentage differences in counts) is presented in Table 1. In 2020, there were 15 550 new diagnoses in the dataset. This represented a drop in incidence by 4772 (31%) cases as compared to the predicted 20 322 (95% CI 19 241–21 403). Similarly, in 2021, although the incidence started to recover, the 17 950 cases were still lower by 3148 (18%) than the estimated 21 098 (95% CI 19 740–22 456) cases that would be diagnosed if the pandemic had not occurred. By 2022, the incidence returned to the levels that would be expected. The difference between 21 975 cases observed in 2022 and 21 874 (95% CI 20 230–23 518) predicted was not statistically significant. Based on the January to July data, we extrapolated that there would be 23 777 new diagnoses in 2023 in the dataset. This is 1289 cases more than the predicted 22 488 (95% CI 20 614–24 363).

The information on demographic characteristics of men at diagnosis by study year is presented in Table 2. There were no differences in demographic characteristics of men diagnosed with prostate cancer by ethnicity and by IMD. However, statistically significant differences were reported by age at diagnosis between the study years. In 2020, the average age at diagnosis was 71.6 (95% CI 71.5–71.8) years. This was higher than the average age in 2019 of 71.3 (95% CI 71.1–71.4) years (*P* = 0.001). The average age at diagnosis increased again in 2021 to 71.8 (95% CI 71.7–72.0) years. This was higher than the 2020 (*P* = 0.035). By 2022, the age at diagnosis dropped to 71.4 (95% CI 71.3–71.5) years, which was statistically significantly lower than 2021 (*P* < 0.001) and dropped again in 2023 to 71.0 (95% CI 70.8–71.1) years (*P* < 0.001). This increase in the average age of men at diagnosis during the pandemic years 2020 and 2021 was generally driven by a ~2% shift from the 65–74 age group to the 75–84 age group.

## Discussion

### Summary and Findings in Context

In this study, we reported a drop in prostate cancer incidence by 31% in 2020 and by 18% in 2021. Taking into account that our dataset represented 40% of the English population, and that in England before the COVID-19 pandemic there were ~44 000 diagnoses per year [18], this drop in incidence represented ~13 640 missed cases in 2020 and ~7920 missed cases in 2021 in England alone. This agreeed with the previously published statistics. There were 38 500 less cancer diagnoses reported in England in 2020 than in 2019 [19]. A third of that, so 12 000 missed cases, was attributed to prostate cancer [15]. This drop in cancer incidence in England has been mirrored across Europe [20] and globally [21], with 1 million cancer diagnoses missed during the pandemic in Europe alone.

The effect of the COVID-19 pandemic was evaluated in this study based on the time trends. This means that any definitive conclusions about the causality were limited and could be biased by other events occurring at the same time. The observed reduction in new diagnoses could have multiple explanatory factors not limited to the COVID-19 pandemic. For example, we observed that in 2018, the incidence of prostate cancer increased by 23% as compared to 2017. This was widely reported and termed in literature as the Turnbull and Fry effect [22]. Two UK public figures promoted prostate cancer awareness, prompting more men to access healthcare, and leading the government to increase prostate cancer funding by £10 million. This was an unprecedented increase, with potential implications for the interpretation of the COVID-19 data. With many men potentially diagnosed early, this could have impacted future years and some decrease attributed to the pandemic could be related to this effect.

The characteristics of men diagnosed with prostate cancer also changed. We observed a shift during the pandemic towards older men. We did not have access to the cancer staging data, but a similar pattern with a shift towards older men [14,15,21] with more advanced cancer stages [14] has been previously reported. The extent to which the delays in cancer diagnosis and shifts towards older men will have negative consequences on patients and cancer services is yet to be understood.

During the early years of the pandemic, there were attempts to project cancer incidence and mortality post pandemic. It was predicted that in 2021 and 2022 there would be more diagnoses of prostate cancer than in the years before the pandemic, to account for the missed cases [23]. We did not observe this to be the case. With incident cases just levelling up to the pre-pandemic level by December 2022, nearly 3 years into the COVID-19 pandemic, the missing 20 000 prostate cancer cases in England have not been accounted for. The prediction also estimated that in 2022–2024 there would be a 17% increase in prostate cancer-related mortality [23]. We also did not observe this. The mortality was stable and overall was not affected by the pandemic. The questions remain, what were the reasons for the reduction in the number of diagnoses, what happened to the people who would have been diagnosed if the pandemic had not occurred, and what impact will this have on their health, quality of life and survival. At present we do not fully understand the consequences of this crisis.

### Strengths and Limitations

The OpenSAFELY-TPP is a population-based and nationally representative dataset of routine healthcare records. The unprecedented size, data quality and completeness of OpenSAFELY is the key strength of this study [16]. The wide time window, from January 2015 to July 2023, and the availability of the near real-time data is another important strength as we were able to investigate the recovery from the effects of the COVID-19 pandemic. To our knowledge this is the first study presenting trends in prostate cancer diagnosis for the period up to the middle of 2023.

The interrupted time series approach for predicting rates and counts during the COVID-19 pandemic based on pre-pandemic trends has an advantage over the approach of simply comparing the COVID-19 period to the same period in pre-COVID-19 years. It provides a less biased estimate of the effect of the pandemic. This is because we account for the long-term trends in healthcare data (e.g., the year-on-year increase or decrease). We used data from 2015 to 2019 to model the trends in the COVID-19 pandemic, which made the prediction of the expected rates more accurate. However, it is important to note that no statistical model is perfect and that the number of missing cases is an estimate and not an exact figure.

The transparent and open research approach is also a strength. All the analytical software and clinical code lists are shared openly and are available for inspection and re-use, providing opportunity for scrutiny, reproduction, and reducing duplicative efforts.

We also note some limitations. This is a dataset based in England only. Therefore, research from other countries is required to understand the global perspective. Lists of clinical codes used to extract data may not be exhaustive and may miss cases. Prostate cancer diagnoses were extracted from primary care, rather than via linkage with cancer registry (the ‘gold standard’ data for cancer). It is therefore possible that some prostate cancer cases were missed or miscoded. However, in the UK, the information about cancer diagnosis is sent to primary care within the hospital discharge letters and primary care is a valid source of these data [24]. We validated the results against other published studies, and they closely align, confirming the validity of the methodology.

## Conclusions

We predicted that 20 000 prostate cancer cases have been missed in 2020 and 2021 in England alone. Although the incidence returned to the pre-pandemic levels by the end of 2022, we show that there has not yet been an increase in incidence to account for the missed cases and that the prevalence of prostate cancer was lower at the end of the study than it would have been if the pandemic had not occurred. We also show a shift in 2020 and 2021 towards diagnosing men in older age. More research is needed to investigate the consequences of this on patients and healthcare systems.

## Figures and Tables

**Table 1:** Prostate cancer incidence by year.

Year	2015	2016	2017	2018	2019	2020	2021	2022	2023 (pro-rata based on January to July)
Monthly rates, per 100 000 adult men	16	16	16	19	17	13	15	18	19
Observed counts	16 765	17 100	17 460	21 465	20 295	15 550	17 950	21 975	23 777
Predicted count (95% CI)						20 322 (19 241–21 403)	21 098 (19 740–22 456)	21 874 (20 230–23 518)	22 488 (20 614–24 363)
Difference, %						–4772 (–31)	–3148 (–18)	101 (0)	1289 (5)
Analysis by region in England									
East Midlands									
Rate	15	15	16	18	18	13	15	18	18
Difference, %						–31	–24	–6	–6
East									
Rate	18	17	17	20	18	14	15	18	19
Difference, %						–31	–18	–2	3
London									
Rate	8	8	9	11	9	7	9	11	11
Difference, %						–35	–5	11	7
North East									
Rate	14	13	14	17	16	13	15	19	21
Difference, %						–29	–14	5	10
North West									
Rate	14	15	15	17	17	12	15	18	21
Difference, %						–32	–11	5	16
South East									
Rate	20	19	20	22	24	15	17	23	22
Difference, %						–44	–33	–2	–11
South West									
Rate	20	19	18	23	20	16	18	20	24
Difference, %						–22	–10	5	17
West Midlands									
Rate	14	14	13	13	12	9	10	13	15
Difference, %						–40	–19	4	19
Yorkshire and The Humber									
Rate	14	14	15	18	16	13	15	18	19
Difference, %						–30	–18	0	2

Statistical significance is estimated using 95% CIs. Statistically significant differences are marked with an asterisk.

**Table 2:** Demographic characteristics of men diagnosed with prostate cancer by year.

Year	2015	2016	2017	2018	2019	2020	2021	2022	2023 (pro-rata based on January to July data)
*P* value by age at diagnosis	n/a	0.274	0.560	0.015	0.151	0.001	0.035	<0.001	<0.001
Average (95% CI)	71.3 (71.2–71.5)	71.4 (71.3–71.6)	71.4 (71.2–71.5)	71.1 (71.0–71.3)	71.3 (71.1–71.4)	71.6 (71.5–71.8)	71.8 (71.7–72.0)	71.4 (71.3–71.5)	71.0 (70.8–71.1)
*P* value by age group	n/a	0.888	0.166	0.002	0.098	0.101	<0.001	<0.001	<0.001
<65 years, *n* (%)	3760 (22)	3795 (22)	3985 (23)	4980 (23)	4720 (23)	3455 (22)	3925 (22)	5250 (24)	6146 (26)
65–74 years, *n* (%)	6850 (41)	6995 (41)	7210 (41)	9045 (42)	8355 (41)	6415 (41)	7065 (39)	8515 (39)	9171 (39)
75–84 years, *n* (%)	4670 (28)	4830 (28)	4755 (27)	5775 (27)	5580 (27)	4400 (28)	5420 (30)	6565 (30)	6943 (29)
≥85 years, *n* (%)	1475 (9)	1510 (9)	1530 (9)	1660 (8)	1660 (8)	1280 (8)	1530 (9)	1650 (8)	1500 (6)
*P* value by ethnicity	n/a	0.009	0.519	0.089	0.108	0.735	<0.001	0.779	0.763
White, *n* (%)	12 950 (96)	13 245 (95)	13 815 (95)	17 240 (96)	16 540 (95)	12 730 (95)	14 700 (94)	18 280 (94)	19 903 (94)
Black, *n* (%)	265 (2)	280 (2)	330 (2)	370 (2)	375 (2)	265 (2)	405 (3)	505 (3)	557 (3)
Asian, *n* (%)	175 (1)	250 (2)	260 (2)	275 (2)	295 (2)	230 (2)	310 (2)	400 (2)	411 (2)
Other (including Chinese and Mixed ethnicities), *n* (%)	130 (1)	135 (1)	145 (1)	160 (1)	190 (1)	140 (1)	195 (1)	265 (1)	266 (1)
Missing, *n* (%)	3230 (19)	3180 (19)	2925 (17)	3425 (16)	2895 (14)	2155 (14)	2335 (13)	2520 (11)	2623 (11)
*P* value by IMD	n/a	0.343	0.002	0.181	0.057	0.284	0.223	0.091	0.252
1 (most deprived), *n* (%)	2050 (12)	2080 (12)	2075 (12)	2480 (12)	2465 (12)	1810 (12)	2225 (13)	2570 (12)	2829 (12)
2, *n* (%)	2655 (16)	2690 (16)	2990 (17)	3515 (17)	3215 (16)	2515 (16)	2900 (16)	3580 (17)	4054 (17)
3, *n* (%)	3920 (24)	3895 (23)	3885 (23)	4855 (23)	4620 (23)	3575 (23)	4110 (23)	4885 (23)	5323 (23)
4, *n* (%)	3970 (24)	4025 (24)	4205 (24)	5270 (25)	4800 (24)	3765 (25)	4240 (24)	5250 (24)	5537 (24)
5 (least deprived), *n* (%)	3910 (24)	4145 (25)	4060 (24)	5080 (24)	4885 (24)	3655 (24)	4145 (24)	5215 (24)	5537 (24)
Missing, *n* (%)	265 (2)	265 (2)	245 (1)	295 (1)	315 (2)	235 (2)	335 (2)	470 (2)	506 (2)

Percentages are calculated from the available data. The proportion of missing data is also reported. P values estimate the statistical significance of year-on-year differences using t-test for means and chi-squared test for proportions. n/a, not applicable.

## Data Availability

Access to the underlying identifiable and potentially re-identifiable pseudonymised electronic health record data is tightly governed by various legislative and regulatory frameworks and restricted by best practice. The data in OpenSAFELY are drawn from General Practice data across England where TPP is the Data Processor. TPP developers initiate an automated process to create pseudonymised records in the core OpenSAFELY database, which are copies of key structured data tables in the identifiable records. These are linked onto key external data resources that have also been pseudonymised via Secure Hash Algorithm (SHA)-512 one-way hashing of NHS numbers using a shared salt. Bennett Institute for Applied Data Science developers and Principal Investigators holding contracts with NHS England have access to the OpenSAFELY pseudonymised data tables as needed to develop the OpenSAFELY tools. These tools in turn enable researchers with OpenSAFELY Data Access Agreements to write and execute code for data management and data analysis without direct access to the underlying raw pseudonymised patient data, and to review the outputs of this code. All code for the full data management pipeline—from raw data to completed results for this analysis—and for the OpenSAFELY platform as a whole is available for review at github.com/OpenSAFELY. Software for data extraction and analysis, and the code lists used to define variables in this study, are shared openly for review and re-use under Massachusetts Institute of Technology (MIT) open licence at: https://github.com/opensafely/ProstateCancerPrevalence. Detailed pseudonymised patient data is potentially re-identifiable and therefore not shared. We rapidly delivered the OpenSAFELY data analysis platform without prior funding to deliver timely analyses on urgent research questions in the context of the global COVID-19 health emergency: now that the platform is established we are developing a formal process for external users to request access in collaboration with NHS England who is the data controller; details of this process are available at OpenSAFELY.org.

## Abbreviations

EHR: electronic healthcare record
IMD: index of multiple deprivation
TPP: The Phoenix Partnership.
